# USP7/HAUSP Promotes the Sequence-Specific DNA Binding Activity of p53

**DOI:** 10.1371/journal.pone.0013040

**Published:** 2010-09-27

**Authors:** Feroz Sarkari, Yi Sheng, Lori Frappier

**Affiliations:** 1 Department of Molecular Genetics, University of Toronto, Toronto, Ontario, Canada; 2 Department of Biology, York University, Toronto, Ontario, Canada; Roswell Park Cancer Institute, United States of America

## Abstract

The p53 tumor suppressor invokes cellular responses to stressful stimuli by coordinating distinct gene expression programs. This function relies heavily on the ability of p53 to function as a transcription factor by binding promoters of target genes in a sequence specific manner. The DNA binding activity of the core domain of p53 is subject to regulation via post-translational modifications of the C-terminal region. Here we show that the ubiquitin specific protease, USP7 or HAUSP, known to stabilize p53, also regulates the sequence-specific DNA binding mediated by the core domain of p53 *in vitro*. This regulation is contingent upon interaction between USP7 and the C-terminal regulatory region of p53. However, our data suggest that this effect is not mediated through the N-terminal domain of USP7 previously shown to bind p53, but rather involves the USP7 C-terminal domain and is independent of the deubiquitylation activity of USP7. Consistent with our *in vitro* observations, we found that overexpression of catalytically inactive USP7 in cells promotes p53 binding to its target sequences and p21 expression, without increasing the levels of p53 protein. We also found that the USP7 C-terminal domain was sufficient for p21 induction. Our results suggest a novel mode of regulation of p53 function by USP7, which is independent of USP7 deubiquitylating activity.

## Introduction

The tumor suppressor p53, often referred to as the guardian of the genome, functions by integrating signals of cellular stress and controlling cell fate [1]. Depending on the nature of stimulus and the extent of cellular stress, p53 orchestrates responses that range from transient cell cycle arrest, allowing for DNA repair and cell survival, to programmed cell death or apoptosis [2]. Aberrant function of a tumor suppressor like p53 would lead to unchecked growth and onset of cancer. It is thus not surprising that inactivation of p53 function through either mutations or interactions with cellular or viral proteins is one of the most common oncogenic events in human cancers [1], [3], [4].

p53 fulfills its tumor suppressive function primarily by acting as a transcription factor. p53 binds DNA as a dimer of dimers in a sequence specific manner to a consensus site comprising of two decamer repeats of 5′-PuPuPuC(A/T)(T/A)GPyPyPy-3′ (where Pu is a purine and Py is a pyrimidine) separated by 0 to 13 base pairs [5]. p53 predominantly activates transcription of target genes, though evidence of transcriptional repression by p53 also exists [6]. A growing body of work has also unearthed a cytosolic and transcription-independent function of p53 [7]. In this role, p53 interacts with anti apoptotic and pro-apoptotic BCL family of proteins and helps bring about permeablization of the outer mitochondrial membrane, which subsequently results in apoptosis. Though cytoplasmic functions of p53 are not strictly dependent on p53 transcription activation, transcriptional regulation by p53 is still tied to the cytosolic functions of p53 since some of the BCL family members are direct transcriptional targets of p53 [7]. The importance of DNA binding and therefore transcriptional control by p53 is further highlighted by the observation that many of the p53 mutations found in tumors are clustered in the DNA binding domain [8] (UMD p53 database 2008_R2; http://p53.free.fr/).

The p53 protein is organized in distinct functional and structural domains. Transcription activation is mediated by the N-terminal transactivation domain (residues 1–70). Residues 94–292 form the DNA-binding domain, which binds DNA in a sequence-specific manner and is also referred to as the core domain. Further downstream is the oligomerization region (residues 320–360) which mediates p53 tetramerization, the functional form of p53 as a transcription factor. The extreme C-terminus of p53 (residues 360–393) forms a lysine and arginine rich basic region and possesses sequence-nonspecific DNA binding activity that is independent of the core DNA binding domain. This region, also known as the regulatory region, was initially thought to negatively regulate the DNA binding activity of the core domain. This notion was based on the observations that deletion and post-translational modifications of the regulatory region or its interaction with an antibody (PAb 421) directed at a C-terminal epitope, lead to an increase in DNA binding by the core domain [9], [10], [11], [12], [13]. It was proposed that these modifications of the C-terminal regulatory domain of p53 induce an allosteric conformational change that switches the core domain from a latent form with low affinity for its DNA binding site to an active form with higher affinity for DNA [9], [14], [15]. These studies however mostly relied on short stretches of naked DNA containing p53-binding sites. The conformational change model was not supported by an NMR-study that showed that full length p53 (latent form) and p53 lacking the C-terminal regulatory domain (active form) were identical in structure [16].

More recent lines of evidence have suggested a positive role for the regulatory region in DNA binding by the core domain. First, a deletion mutant lacking the C-terminal region (p53Δ30) shows weaker DNA binding ability than WT p53, when longer molecules of DNA are used [17]. Second, efficient recognition of target sites in circular DNA or stemloop structures requires the C-terminal region of p53 [18], [19]. Third, it was shown that the C-terminal region of p53, through its nonspecific DNA binding activity, helps p53 slide along stretches of DNA [20], [21]. Linear diffusion along DNA allows the p53 core domain to sample sequences and find its target sites. Thus, the p53 C-terminus positively contributes to sequence specific DNA binding by the p53 core domain through mechanisms that are not fully understood.

An important regulator of p53 function is the herpesvirus associated ubiquitin specific protease, HAUSP or USP7, which deubiquitylates p53 and protects it from proteasome-mediated degradation [22]. Deletion analyses have shown that the C-terminal regulatory region of p53 (residues 351–382) binds USP7 and that the N-terminal domain (residues 53–208) of USP7 is sufficient for this interaction [23], [24]. Crystal structures of the USP7 N-terminal domain showed that it is a TRAF domain and that a groove on its surface forms interaction with p53 and other targets [25], [26], [27]. USP7 was originally identified as a binding partner of the ICP0 protein from herpes simplex virus [28] and was shown to interact with another herpesvirus protein, EBNA1 of Epstein-Barr virus (EBV) [29]. We have shown that EBNA1 can alter cellular processes, including p53 function, through its interaction with USP7 [25], [30]. However, EBNA1′s more traditionally known functions rely on its DNA binding activity to mediate replication and segregation of the EBV genome and transactivation of viral genes [31]. Interestingly, we have shown that USP7 stimulates the DNA binding activity of EBNA1 and is important for transcriptional activation by EBNA1 at the latent origin of EBV replication [32]. However it was unclear whether this ability of USP7 to stimulate DNA binding activity was only relevant for EBNA1 or might also apply to other USP7 targets.

Given that USP7 binds the C-terminal domain of p53 [24] and that this domain regulates DNA binding by the p53 core domain, we asked whether USP7 affects the DNA binding activity of p53 and downstream p53 functions. In this study, we discuss observations that support a role of USP7 in regulating p53 DNA binding. This provides a novel aspect of p53 regulation by USP7, since it is independent of p53 deubiquitilyation.

## Results

### Effect of USP7 on DNA Binding by p53

**Figure 1:**
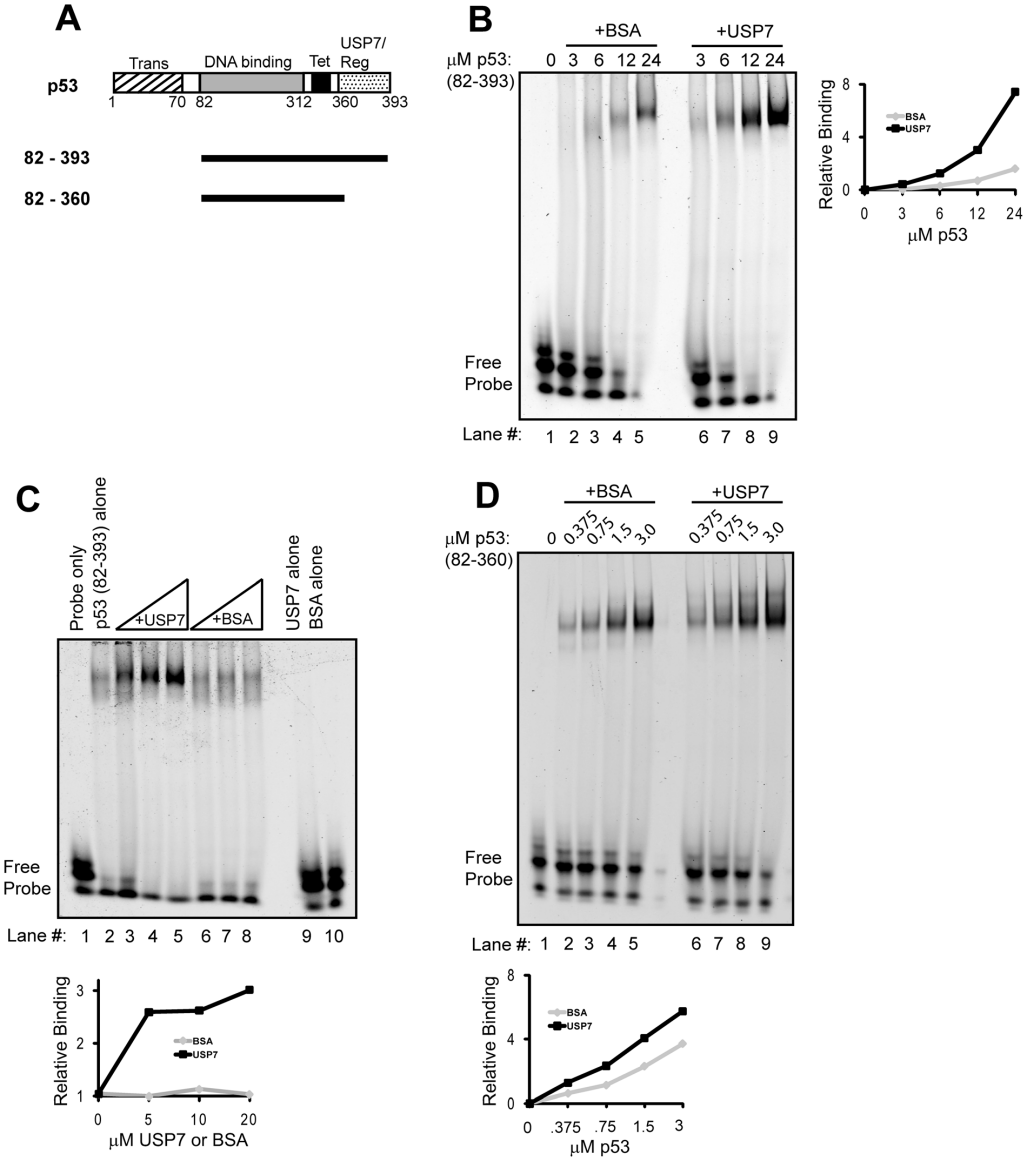
Effect of USP7 on the DNA binding activity of p53 in vitro. (A) Schematic representation of the p53 proteins used in this study showing the transactivation (Trans), DNA binding core, tetramerization (Tet) and USP7-binding and regulatory (USP7/Reg) regions. (B) EMSA showing titration of latent p53_82–393_ in the presence of 20 µM of BSA negative control (lanes 2–5) or 20 µM USP7 (lanes 6–9). (C) EMSA performed with fixed amount (12 µM) of p53_82–393_ and with 5 µM, 10 µM or 20 µM of USP7 (lanes 3–5) or BSA (lanes 6–8). Incubation of 20 µM of USP7 alone or BSA alone with labeled probe in the absence of p53 is also shown (lanes 9 and 10). (D) EMSA showing titration of active p53_82–360_, in the absence (lanes 2–5) or presence of USP7 (lanes 6–9). Quantification of the discreet shifted bands for parts B,C and D are shown in the graphs, with USP7 in black and BSA in grey.

To assess the effect of USP7 on p53 DNA binding, we conducted electrophoretic mobility shift assays (EMSAs) using a Cy-5 labeled consensus p53 binding sequence as a probe and a version of p53 spanning the core DNA binding and the C-terminal regulatory regions (p53_82–393_) but lacking the transactivation domain, (Figure 1A). Purified p53_82–393_ was incubated with the labeled DNA in the presence of USP7 or BSA as a negative control. This version of p53 is termed the latent form since in EMSAs it exhibits decreased sequence-specific DNA binding, as characterized by the smearing of DNA-protein complexes and, at high protein concentrations, a small amount of a discreet band in the shifted DNA probe (see Figure 1B, lanes 2–5 and Figure 1C lanes, 6–8). In contrast, in the presence of USP7, p53_82–393_ formed DNA complexes that migrated as distinct bands as is characteristic of sequence-specific DNA binding (Figure 1B, lanes 6–9 and Figure 1C lanes 3–5). Experiments were performed both by titrating p53 with a fixed amount of excess USP7 (Figure 1B) and by incubating a fixed amount of p53 with increasing amounts of USP7 (Figure 1C), with similar results. The latter experiment showed that USP7 had a dose-dependant stimulatory effect on the DNA binding ability of p53_82–393_ while the BSA negative control had little to no effect (Figure 1C). Neither USP7 nor BSA detectably bound the DNA probe under these conditions, even at the highest concentrations (Figure 1C, lanes 9 and 10). These results show that USP7 stimulates the DNA binding activity of p53.

### USP7 Stimulates p53 DNA Binding Through Interactions with the p53 C-terminal Regulatory Region

To test whether USP7 binding was responsible for the stimulatory effect on sequence-specific DNA binding by p53, we conducted EMSAs using another version of p53, p53_82–360_ (Figure 1A), which differs from p53_82–393_ in that it lacks the C-terminal regulatory region responsible for both USP7 binding [23] and nonspecific DNA binding. This version is termed the active form of p53 as it lacks autoinhibition from the C-terminal region. As expected, p53_82–360_ efficiently binds DNA, at concentrations much lower than that used for latent p53, as indicated by the distinct shifts in the mobility of the DNA probe (Figure 1D, lanes 2–5). While, the active p53_82–360_ binds better than the latent form, p53_82–393_, its DNA binding was hardly affected by USP7 (Figure 1D, lanes 6–9), indicating that USP7 acts through a specific interaction with the p53 regulatory region.

### USP7 C-terminal Sequences Stimulate p53 DNA Binding

**Figure 2:**
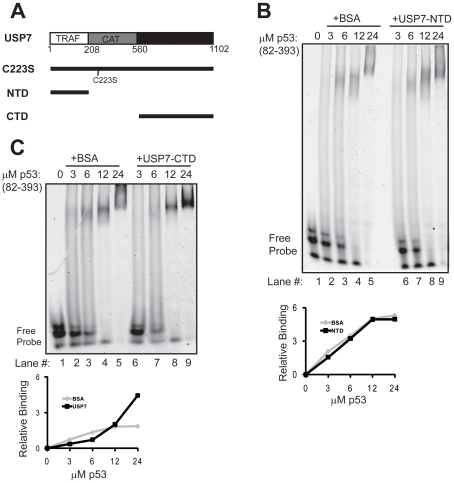
Effects of USP7 NTD and CTD on p53 DNA binding. (A) Schematic representation of the USP7 proteins used in this study showing the N-terminal (NTD) or TRAF domain, central catalytic (CAT) and C-terminal (CTD) domains. The position of the C223S point mutation that inactivates the catalytic domain is also shown. (B–C) EMSAs showing titration of latent p53_82–393_, in the presence or absence of 20 µM USP7-NTD (B) or 20 µM USP7-CTD (C). Quantification of the discreet shifted bands are shown in the graphs, with USP7 in black and BSA in grey.

It has been shown that the N-terminal domain of USP7 (USP7-NTD) is sufficient to interact with p53 [24]. Therefore we examined whether the interaction mediated by USP7-NTD (shown in Figure 2A) was sufficient to stimulate the DNA binding activity of p53. To this end, we performed EMSAs with latent p53_82–393_ in the presence and absence of USP7-NTD. Surprisingly the USP7-NTD did not stimulate sequence-specific DNA binding by p53_82–393,_ as the p53-DNA complexes migrated as smears rather than discreet bands both in the presence and absence of the USP7-NTD (Figure 2B). This suggests that interactions occur between p53 and USP7 regions other than the USP7-NTD, which is responsible for the stimulatory effect of USP7 on p53 DNA binding.

USP7 C-terminal regions downstream of the catalytic domain are also known to mediate some protein interactions [33], [34] including weak interactions with p53 [24]. Therefore we tested whether the USP7-CTD (amino acids 560–1102 as shown in Figure 2A) could account for the effect of USP7 on p53 DNA binding by assaying the DNA binding activity of p53_82–393_ in the presence and absence of this USP7 domain (Figure 2C). Similar to what we observed with full-length USP7, the USP7-CTD stimulated sequence-specific DNA binding by p53, as compared to the BSA control, suggesting that it is largely responsible for the p53-USP7 interaction that results in increased p53 sequence-specific DNA binding.

### USP7 Promotes p53 DNA-binding *in vivo*

**Figure 3:**
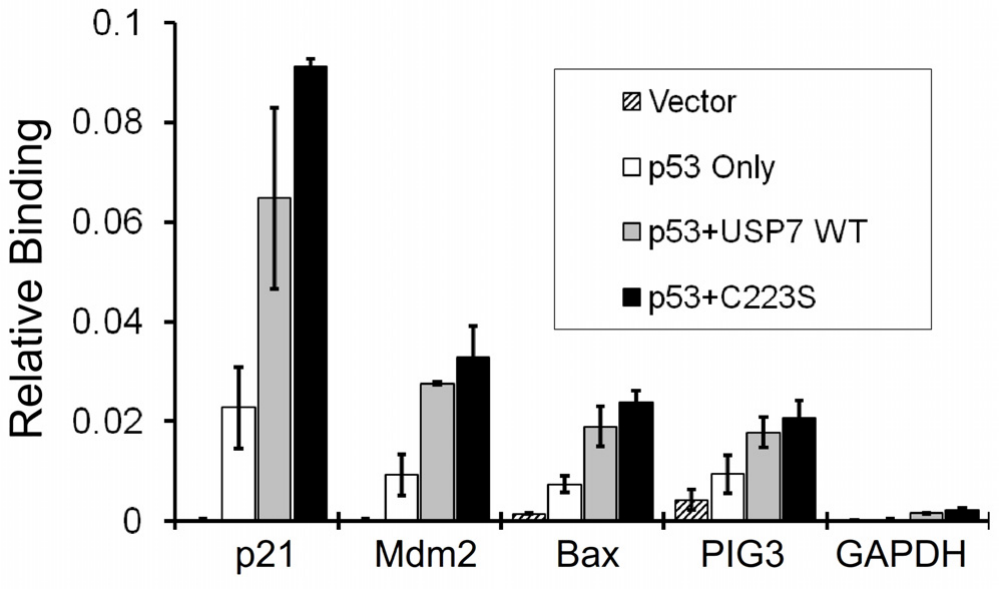
USP7 promotes p53 DNA-binding in vivo. H1299 cells were either transfected with an empty plasmid (Vector) or transfected with a p53-expressing plasmid alone or in combination with constructs expressing either WT myc-tagged USP7 (+USP7 WT) or myc-tagged C223S (+C223S). p53 occupancy of various promoters in transfected cells was measured by chromatin immunoprecipitation using a p53 antibody and Q-PCR of the target sequences indicated. GAPDH was used a negative control region for Q-PCR. Results were normalized to p53 levels determined for each sample by Western blotting (see Figure 4B for an example) to adjust for any small variations in p53 levels.

We next investigated whether USP7 stimulates p53 DNA-binding in cells. To this end, p53-negative H1299 cells were transfected with a plasmid expressing p53 alone, with or without a plasmid expressing WT USP7 or a catalytically inactive mutant of USP7, C223S. C223S binds p53 but does not stabilize it due to the lack of ubiquitin cleavage activity and has been shown to destabilize p53 through a dominant negative effect [24]. The use of this USP7 mutant ensured that any stimulation of p53 function was not due to increased levels of p53. Promoter occupancy by p53 was measured by chromatin immunoprecipitation (ChIP) using p53 antibody and quantitative PCR of various p53 target sequences (p21, Mdm2, Bax and PIG3) 24 hours post transfection. p53 immunoprecipitates were enriched for all p53 target promoters tested, with the highest level of binding detected at the p21 promoter (Figure 3). Little to no DNA was recovered for the negative control GAPDH region. Consistent with our *in vitro* results, co-expression of USP7 or C223S stimulated binding of p53 to all the p53-responsive promoters tested and not the non-specific GAPDH control. The finding that C223S stimulated p53-DNA interactions in cells to a similar degree as WT USP7 indicates that this stimulation is independent of p53 stabilization.

### USP7 can Stimulate p53 Function Independent of its Catalytic Activity

**Figure 4:**
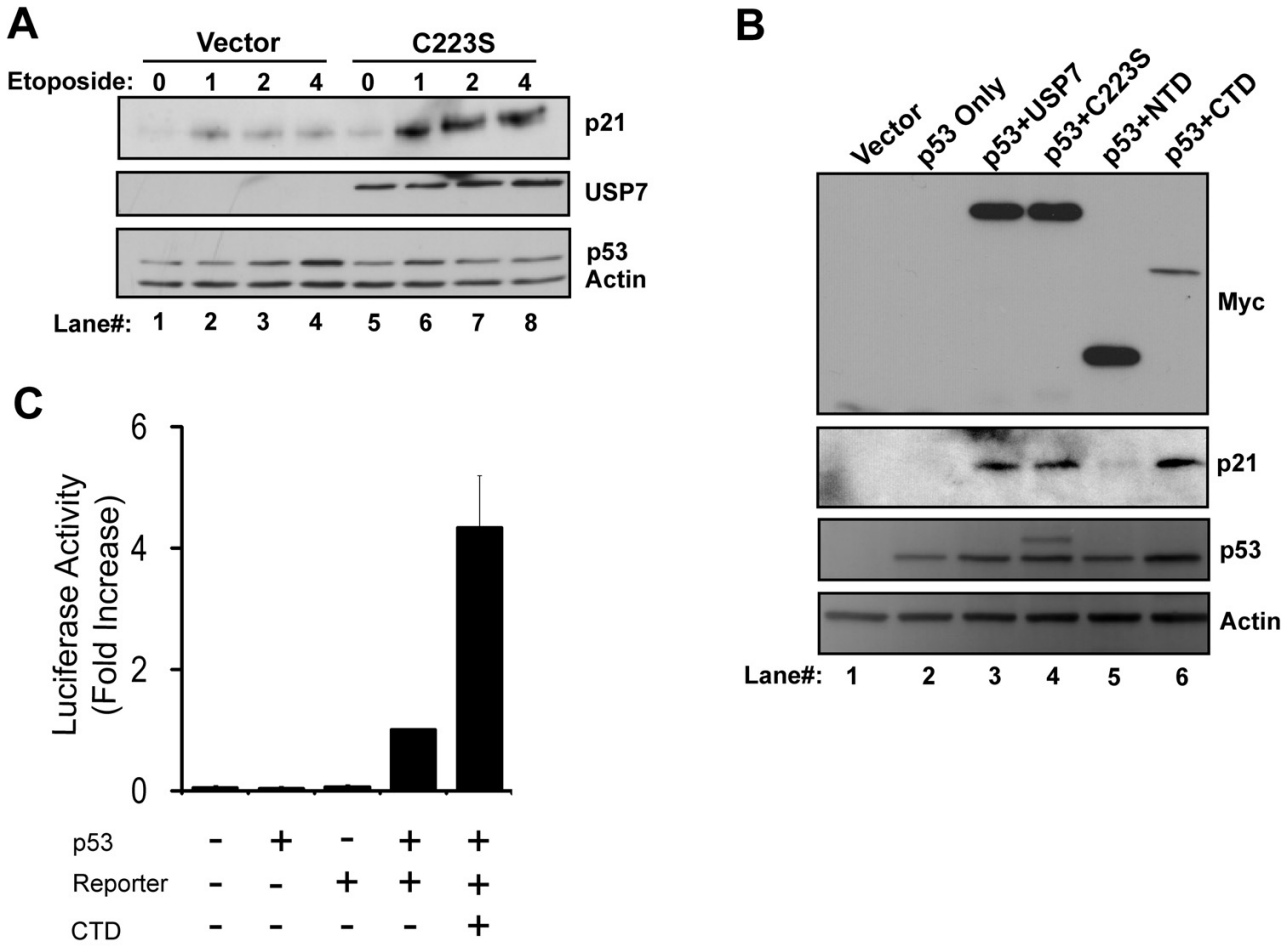
Catalytically inactive USP7 stimulates p53 function. (A) U2OS cells were transfected with an empty vector or vector expressing the myc-tagged C223S mutant of USP7 and treated with etoposide for 0, 1, 2 or 4 hours as indicated. Equal amounts of cell lysates were analyzed for protein expression by western blotting using the indicated antibodies where actin is the loading control. (B) H1299 cells were either transfected with an empty plasmid (Vector) or transfected with a p53-expressing plasmid alone or in combination with constructs expressing myc-tagged USP7 and USP7 mutants as indicated. 24 hours post-transfection, cells were lysed and protein levels were measured by Western blotting using the antibodies indicated. The higher p53 band in lane 4 corresponds to monoubiquitylated p53 which becomes apparent due to the dominant-negative effects of C223S [24]. (C) H1299 cells were transfected with plasmids expressing p53 and myc-tagged USP7-CTD and a luciferase reporter construct in the combinations indicated. 48 hours post-transfection, cells were lysed and luciferase activity was quantified. The results are shown relative to the p53 plus reporter sample for three independent experiments.

The results above show that USP7 promotes binding of p53 to its target DNA. Since the most striking results were obtained for the p21 promoter, we focused further studies on p53 function on inductions of the p21 gene. First, we tested whether USP7 could stimulate p53 function in a ubiquitin-independent manner during conditions of cellular stress such as DNA damage. To this end we measured induction of p21 expression in U2OS cells, which express wild type p53. p21 protein levels are normally low, however in response to DNA damage, p53 activates transcription of the gene encoding p21 and hence increases p21 protein levels. U2OS cells were transfected with either an empty vector or a vector expressing C223S. Since C223S expression does not stabilize p53, the use of this USP7 mutant ensured that any stimulation of p53 function was not due to increased levels of p53. Etoposide treatment of U2OS cells transfected with the empty vector led to stabilization of p53, which was accompanied by accumulation of p21 (Figure 4A, compare lane 1 to 2–4). p53 stabilization was diminished in C223S-transfected cells, consistent with its dominant negative effects (Figure 4A; compare lanes 3 and 4 to lanes 7 and 8). However expression of p21 was increased by C223S both before and, more dramatically, after etoposide treatment (Figure 4A, top panels, compare lanes 1–4 to 5–8). These observations suggest that after induction of DNA damage, in addition to stabilizing p53, USP7 can also stimulate p53 DNA-binding and serve a dual role in p53 regulation under conditions of cellular stress.

Next we tested whether USP7 enhanced p53 function by examining the induction of p21 in H1299 cells after transfection of a p53-expressing plasmid alone or in combination with a plasmid expressing either WT USP7 or a USP7 mutant (Figure 4B). At the levels of p53 expressed, little to no p21 expression was observed in the p53 only sample (Figure 4B, lane 2). Consistent with the ChIP results, coexpression of WT USP7 or C223S led to induction of p21 (lanes 3 and 4). We also tested the effect of the USP7-NTD and USP7-CTD on p21 expression. The USP7-CTD stimulated p21 expression, whereas minimal effect was seen with USP7-NTD (lanes 5 and 6). These results are consistent with the *in vitro* observation that the CTD of USP7 is sufficient to stimulate p53-DNA binding, and together these observations suggest that the stimulation of p53 DNA-binding by USP7 leads to enhanced p53 function.

The observations above show that the USP7-CTD is sufficient to promote DNA binding by p53 *in vitro* and p21 expression in cells, suggesting that the USP7-CTD contributes to transcriptional activation by p53. To test this more directly, we cotransfected H1299 cells with a reporter plasmid in which expression of the luciferase gene is under control of the p21 promoter, along with a p53-expression plasmid or corresponding empty plasmid (Figure 4C). The reporter construct alone or the p53 expression vector alone showed minimal to no luciferase activity, while expression of p53 with the reporter construct gave luciferase activity above background. However coexpression of USP7-CTD with p53 resulted in a 5-fold increase in luciferase activity, indicating that the USP7-CTD can stimulate p53 transactivation from the p21 promoter.

## Discussion

The DNA-binding ability of p53 is critical to its function as a transcription factor and thus as a tumor suppressor. The significance of sequence-specific DNA binding for p53 tumor suppressor function is highlighted by the substantial number of tumor-associated mutations in the core DNA-binding domain [35]. An important determinant of DNA binding by the core domain is the autoregulation of this activity by the C-terminal regulatory domain of p53. The C-terminal domain is heavily modified post-translationally and these modifications affect the ability of p53 to bind DNA [36], [37]. Here we propose that binding to the ubiquitin specific protease, USP7, is yet another means of regulating this property of p53.

Full length p53, with the core DNA binding domain and the C-terminal domain intact, is referred to as the latent form, since it shows poor sequence-specific DNA binding in *in vitro* binding assays. This effect is attributed to autoinhibition of sequence-specific DNA binding by the C-terminal domain due to increased DNA sliding [20], [21]. Incubation of latent p53 with USP7 in EMSAs stimulated sequence-specific DNA binding by latent p53, suggesting that USP7-binding can reverse this autoinhibition. DNA binding by a C-terminal deletion mutant of p53, p53_82–360_, which lacks the USP7 binding sequences, was not stimulated by USP7, suggesting that binding to USP7 is required for this effect. The observation that USP7 stimulates sequence-specific DNA binding by p53 through interaction with the C-terminal regulatory region of p53 further support the notion that this region of p53 is a positive regulator of p53 DNA-binding and p53 function [20]. Since the p53 regulatory region contributes to DNA interactions by increasing the sliding of p53 on DNA and such sliding has been suggested to result in decreased detectable binding to short DNA fragments (such as used in our *in vitro* studies), it is likely that USP7 interactions with p53 C-terminal sequences result in decreased DNA sliding [20].

The USP7-NTD, which is sufficient to bind p53, had no obvious effect on the DNA-binding activity of p53. While the interaction of p53 with the USP7-NTD is important for deubiquitylation and stabilization of p53, the results here suggest that interactions mediated by other regions of USP7 are important for the effect on p53 DNA binding. In support of this assumption, we found that the USP7-CTD can stimulate DNA binding by latent p53. The results suggest that the USP7-CTD can also mediate p53 interactions and that this interaction is largely responsible for stimulating the sequence-specific DNA binding activity of p53. This is in keeping with initial reports by Li et al [24] that weak interaction between p53 and the USP7 C-terminal region (637–1102) could be detected in GST pull-down assays. It should be noted that, the USP7-CTD is also known to mediate interactions with other proteins including FOXO [34] and the ICP0 protein of herpes simplex virus [33].

This investigation was prompted by our previous observation that USP7 stimulated the DNA binding activity of the EBV EBNA1 protein [32]. There are similarities and subtle differences in how USP7 contributes to the DNA binding activity of these two very different proteins. The USP7-NTD has been shown to be sufficient for binding both p53 and EBNA1 [25], [33]. However, while the USP7-NTD can stimulate DNA binding by EBNA1, it had no effect on p53 DNA binding. For EBNA1, the USP7-NTD did not stimulate DNA binding to the same degree as the full length USP7, suggesting that the USP7-CTD also contributes to this effect. Therefore, for both EBNA1 and p53, the data suggest that USP7 regions other than the NTD can contribute to interactions that stimulate DNA binding (albeit to differing degrees). Another difference in how USP7 affects DNA binding by EBNA1 and p53 may be in the degree to which it remains associated with the DNA complex. USP7 forms a ternary complex with EBNA1 on DNA and EBNA1 can recruit USP7 to EBNA1 binding sequences in the EBV genome where it affects histone H2B ubiquitylation [32]. This ternary complex is evident *in vitro* by the decreased mobility or supershift of EBNA1-DNA complexes by USP7 in EMSAs [32]. In contrast, p53-DNA complexes migrated similarly in the presence or absence of USP7 (compare shifted bands in Figure 1B), suggesting that USP7 does not remain stably associated with DNA-bound p53. However, this merits further investigation, particularly *in vivo* where the interaction might be stabilized by the presence of additional proteins and/or DNA sequences.

Additionally, we showed that USP7 overexpression consistently promoted p53 binding to several p53 response elements in cells. Interestingly, the catalytically inactive USP7, C223S, which does not stabilize p53, promoted p53 DNA-binding in cells just as well or better than WT USP7. In line with these results, we found that overexpression of C223S, leads to increased p21 levels compared to control cells before and after etoposide treatment, without increasing p53 levels. This is consistent with stimulation of p53 binding to the p21 promoter resulting in enhanced p21 expression. More detailed analysis of the effect of USP7 mutants on p53-dependent p21 expression in cells revealed that the USP7-NTD, shown previously to bind p53, had negligible effect on p21 expression, whereas the USP7-CTD was sufficient to promote p53-dependent p21 expression. These results are consistent with our *in vitro* observations that the USP7-NTD does not stimulate p53-DNA binding and that this stimulation is mediated by the USP7-CTD. Taken together, our results show that USP7 can promote p53 function in a manner that is independent of the interaction through the USP7-NTD and deubiquitylation by the catalytic domain. On that note, we have recently shown that USP7 promotes the degradation of PML proteins (whose gene is activated by p53) by a mechanism that is independent of its catalytic activity [38]. Clearly the role of USP7 in regulating the p53 pathway is more complicated than its previously established role as a deubiquitylating enzyme.

## Materials and Methods

### p53 and USP7 Constructs and Purification

Constructs expressing p53 mutants for purification and the subsequent purification of p53 proteins are described previously [16]. USP7 proteins for *in vitro* studies were expressed and purified as described by Holowaty et al [29]. To generate the pCANmycUSP7 plasmid use for expression in human cells, USP7 cDNA was PCR amplified from the pET3a-USP7 plasmid (a gift from Roger Everett). The amplified fragment was ligated into HindIII and XbaI sites of the pcDNA3.1-derived plasmid, pCANmyc. pCANmycC223S plasmid was generated by QuickChange mutagenesis of pCANmycUSP7 using the following primers: 5′CAGGGAGCGACTTCTTACATGAACAGCCTG3′ and 5′CAGGCTGTTCATGTAAGAAGTCGCTCCCTG3′. USP7 NTD and USP7 CTD fragments were generated by PCR-amplification of the sequences encoding these domains from pCANmycUSP7 using the primers 5′CGCCGCAAGCTTCCGAAAAAAAAAAAACGCAAAGTGATGAACCACCAGCAGCAGC 3′ and 5′ CCGGGATCCTCACTTTGAATCCCACGCAACTCC 3′ for the NTD and 5′CGCCGCAAGCTTCCGAAAAAAAAAAAACGCAAAGTGGAAGCCCATCTCTATATGCAAG 3′ and 5′GCGGGATCCTCAGTTATGGATTTTAATGGCC 3′ for the CTD. The sequence coding for the SV40 T antigen nuclear localization signal was included in the 5′ primers to generate an in-frame NLS at the N-terminus of each domain. Amplified fragments were ligated into pCMVmyc [39] between HindIII and BamHI sites.

### Electrophoretic Mobility Shift Assays (EMSAs)

The labeling of DNA double stranded probes and EMSAs were performed according to Ayed et al [16]. Briefly, p53 was incubated with either BSA or USP7 on ice for 5 minutes prior to incubation with 8 pmoles of Cy-5 Dye labeled DNA double stranded probe (GGACATGCCCGGGCATGTCC). Protein-DNA mixes were further incubated at room temperature for 10 minutes in the presence of 1 µg salmon sperm competitive DNA and total reaction volume was brought up to 20 µL using reaction buffer (20 mM Tris.Cl pH 8.0, 200 mM NaCl). Samples were resolved on 5% polyacrylamide gels at 4°C at 100 V. Gels were scanned using a Typhoon 9400 scanner (Amersham) and analyzed using the ImageQuant 5.0 software. Sequence-specific DNA binding was quantified by determining the amount of Cy-5 in a box containing the discreet shifted band and in the same-sized box at the same position in each lane.

### Western Blots

For Figure 4A, U2OS cells in 10 cm dishes at 80% confluency were transfected with 10 µg pCANmyc or pCANmycC223S using Lipfectamine 2000 (Invitrogen). 24 hours post transfection, cells were either left untreated or treated with 10 µg/mL of etoposide for 1, 2 and 4 hours. Cells were harvested and lysed in 9 M urea, 5 mM Tris.Cl pH 6.8, sonicated briefly and subjected to centrifugation for 1 minute at 15,000 rpm in a microcentrifuge. 50 µg of total protein was subjected to SDS-PAGE and transferred to PVDF membrane (Amersham). For Figure 4B, H1299 cells in 10 cm dishes at 80% confluency were transfected using Lipfectamine 2000 (Invitrogen) with 20 ng of pCDNA3.1-p53 [40] and either 10 µg of empty vector (pCMV-myc) or 10 µg of pCANmyc plasmid expressing WT USP7, C223S, USP7-CTD or USP7-NTD. 24 hours post- transfection cells were lysed in RIPA buffer (20 mM Tris pH 8.0, 150 mM NaCl, 1% NP40, 0.1% Sodium Deoxycholate, 1 mM PMSF) containing protease inhibitor cocktail (Sigma, P8340) and clarified by centrifugation at 15,000 rpm at 4°C. For the p21 blot, 60 mg of total protein was subjected SDS-PAGE and western blotting, whereas 25 µg was used for all other blots. Membranes were blocked in blocking buffer (5% milk in PBS (137 mM NaCl, 2.7 mM KCl, 0.01 mM Na_2_HPO_4_, 1.4 mM KH_2_PO_4_, pH 7.4)). Primary antibodies used were R2B2 for USP7 [29], DO-1 for p53 (Santa Cruz), Ab-1 for Actin (Calbiochem), antibody 187 for p21 (Santa Cruz, sc-817) and antibody A-14 for c-myc (Santa Cruz, sc-789). After primary antibody incubation, membranes were washed in PBS with 0.1% Tween 20 (PBS-T) then incubated with the secondary antibodies goat anti mouse-HRP (Santa Cruz, SC-2055) or goat anti-rabbit-HRP (Santa Cruz, SC-2004). Following washes in PBS-T, blots were developed using chemiluminescence ECL reagent (Perkin Elmer).

### Chromatin Immunoprecipitation (ChIP)

H1299 cells in 10 cm dishes at 80% confluency were transfected with 4 µg of a plasmid expressing p53, 13 µg of vector expressing USP7 or C223S, or an empty vector. 24 hours post-transfection, cells were fixed with 1% formaldehyde, lysed in RIPA buffer containing protease inhibitor cocktail (Sigma, P8340) and sonicated briefly to shear the DNA. Clarified lysates were precleared with Protein A/G beads (Santa Cruz, SC-2003) and normal mouse IgG (Santa Cruz, SC-2343) prior to immunoprecipitation with p53 DO1 antibody (Santa Cruz). Protein cross links were reversed in the immunoprecipitated DNA by incubating at 65°C for 16 hrs. DNA was purified using QIAquick Gel Extraction Kit (Qiagen, 28704) and analyzed by quantitative RT-PCR using LightyCycler 480 DNA SYBR Green I Master (Roche, 04707516001) and a Rotorgene Q-PCR system (Corbett Research). Primers used for quantification were as follows: p21 (Forward: 5′CTGGACTGGGCACTCTTGTC 3′, Reverse: 5′CTCCTACCATCCCCTTCCTC 3′); Mdm2 (Forward 5′GGATTGGGCCGGTTCAGTGG 3′, Reverse 5′GCGTCCGTGCCCACAGGTC 3′); BAX (Forward 5′TATCTCTTGGGCTCACAAG 3′, Reverse 5′ACTGTCCAATGAGCATCTCC 3′); PIG3 (Forward 5′GATCCCAGGACTGCGTTTTGCC 3′, Reverse 5′GGGAACGAGACCCAACCTCTTG 3′) and GAPDH (Forward, 5′TGTTGCCATCAATGACCCCTT 3′ Reverse 5′CTCCACGACGTACTCAGCG 3′). Lysates used for chromatin immunoprecipitation were also subjected to SDS-PAGE and western blotting using antibodies indicated in the results section. ChIP signal was normalized to p53 levels as determined by Western blots developed using the ECL Plus system (GE Amersham) and quantified on a Typhoon Imaging scanner using ImageQuant 5.0 software.

### Luciferase Assay

H1299 cells in 6 cm dishes were grown to 80% confluence and transfected with 0.5 µg of a plasmid containing the luciferase reporter gene fused to p53 specific sequences from the p21 promoter (p21-Luc) [41], 0.01 µg pCDNA3.1-p53 expressing p53 [40] (both plasmids kindly provided by Dr Sam Benchimol) and 4 µg of a either plasmid expressing the USP7-CTD or empty plasmid. 24 hours post-transfection, cells were moved to 10 cm dishes. Cells were harvested 48 hours post-transfection and processed for luciferase assay according to the Promega Luciferase Assay System (E1500). Luciferase activity was measured by the Molecular Devices Spectramax M2E and analyzed by using the Softmax Pro software v5.0.1. Results from at least 3 independent experiments are reported.
